# Early-life exposure to traffic-related air pollution and child anthropometry

**DOI:** 10.1097/EE9.0000000000000061

**Published:** 2019-08-29

**Authors:** Clara G. Sears, Catrina Mueller-Leonhard, Gregory A. Wellenius, Aimin Chen, Patrick Ryan, Bruce P. Lanphear, Joseph M. Braun

**Affiliations:** a Department of Epidemiology, Brown University, Providence, Rhode Island; b Department of Environmental Health, University of Cincinnati College of Medicine, Cincinnati, Ohio; c Department of Pediatrics, University of Cincinnati, College of Medicine, Cincinnati, Ohio; d Division of Biostatistics and Epidemiology, Cincinnati Children’s Hospital Medical Center, Cincinnati, Ohio; e Faculty of Health Sciences, Simon Fraser University, Burnaby, British Columbia, Canada.

**Keywords:** Birthweight, Body mass index, Child health, Gestation, Obesity, Traffic-related air pollution

## Abstract

**Background:**

Early-life exposure to traffic-related air pollution may decrease fetal growth and increase childhood obesity risk. Our objective was to evaluate the relationship of early-life exposure to traffic-related air pollution with birthweight in term newborns and obesity at age 7–8 years in two prospective birth cohorts in Cincinnati, OH (the Health Outcomes and Measures of the Environment [HOME] Study and Cincinnati Childhood Allergy and Air Pollution Study [CCAAPS]).

**Methods:**

We estimated elemental carbon attributable to traffic (ECAT) exposure at residential addresses during pregnancy with a validated land use regression model. We assessed birthweight among term infants using birth records or parent report (HOME Study n = 333 and CCAAPS n = 590). We measured children’s weight and height at 7–8 years and calculated age- and sex-specific body mass index (BMI) z-scores (HOME Study n = 198 and CCAAPS n = 459). Using multivariable linear regression, we estimated the difference in term birthweight and BMI z-score per interquartile range (IQR) increase in ECAT concentrations in each cohort separately and in the pooled sample.

**Results:**

In adjusted models, ECAT exposure was not associated with lower birthweight (pooled sample β: 30 g; 95% CI = −6, 66) or with higher BMI z-score (pooled sample β: −0.04; 95% CI = −0.15, 0.08). Infant sex modified the association between ECAT and birthweight (*P* = 0.05). Among male newborns, higher ECAT concentrations were associated with higher birthweight (β: 61 g; 95% CI = 9, 113), but we observed no association among female newborns (β: −9 g; 95% CI = −58, 41).

**Conclusions:**

In contrast to some prior studies, early-life traffic-related air pollution exposure was not associated with lower birthweight or increased childhood adiposity in these two cohorts.

## Abstract 2

Supplemental Digital Content is available in the text.

**What this study adds**

Our results add to the current research by using a novel marker of traffic-related air pollution, which is sensitive to diesel traffic, to characterize the association of traffic-related air pollution exposure with birthweight and child adiposity.

## Introduction

Childhood obesity, a serious public health concern, has several risk factors, including genetics and socioeconomic characteristics, and exposure to environmental obesogens.^1–3^ One obesogen is ambient air pollution exposure, which is associated with weight status and patterns of weight gain throughout gestation, infancy, and childhood.^4–15^ Pre- and postnatal exposure to ambient and traffic-related air pollution has been shown to contribute to low birthweight and fetal growth restriction, greater infant weight gain, and excess childhood adiposity.^6–8,10,12–14,16–19^ Infants born low birthweight (<2,500 g) have an increased risk for infant mortality and cardiovascular disease and diabetes in adulthood.^20–23^ Furthermore, excess childhood adiposity is associated with adulthood obesity which increases mortality risk.^24–26^

Recent analyses suggest that the potential effect of traffic-related air pollution exposure on obesity risk varies for infants and children living near freeways versus nonfreeways, leading researchers to hypothesize that specific traffic patterns and mixtures of vehicular exhaust may be independently associated with obesity risk.^16,27^ Identifying those vehicle types and roadway designs that present the greatest public health threat can guide effective intervention strategies.

Diesel traffic, which occurs at higher volumes on freeways, generates a higher concentration of fine and ultrafine particulates compared with gasoline engines, potentially posing a greater threat to the developing fetus and child’s health.^28,29^ Using a previously validated diesel source profile and land use regression model, we estimated a sensitive measure of traffic-related air pollution exposure from diesel combustion, elemental carbon attributable to traffic (ECAT) sources, across different roadway classifications in the city of Cincinnati.^30^ Compared with elemental carbon exposure estimates used in previous studies, this novel ECAT measure may provide a better estimate of exposure to the hazardous fine particulates generated by diesel engines. We used this measure to investigate the relationship of residential traffic-related air pollution exposure during early life with birthweight and childhood adiposity at age 7–8 years in the Health Outcomes and Measures of the Environment (HOME) Study and Cincinnati Childhood Allergy and Air Pollution Study (CCAAPS). We hypothesized that higher residential ECAT exposure during early life is associated with lower birthweight and higher childhood adiposity at school age.

## Methods

### Study participants

From March 2003 to January 2006, we recruited HOME Study participants from nine prenatal care clinics associated with three delivery hospitals in the greater Cincinnati, OH metropolitan area.^31^ To be eligible, women had to be living in the study region, 16 ± 3 weeks gestation, 18 years of age or older, residing in a home built in 1978 or prior, not living in a mobile or trailer home, human immunodeficiency virus negative, not taking medications for seizures or thyroid disorders, planning to continue prenatal care and deliver at the collaborating clinics and hospitals, planning to remain in the greater Cincinnati area for the next year, and fluent in English.^31^ Additionally, women could not have a diagnosis of any of the following disorders: diabetes, bipolar, schizophrenia, or cancer that resulted in radiation treatment or chemotherapy. Approval for the study was obtained from the institutional review boards of Cincinnati Children’s Hospital Medical Center and the participating delivery hospitals. Women provided written informed consent after study protocols were explained by trained research assistants.

We recruited CCAAPS study participants from the greater Cincinnati area using birth certificate data from 2001 to 2003. To be eligible, newborns had to reside at their time of birth either <400 or >1,500 m from a major roadway (defined as >1,000 trucks daily), be >35 weeks gestation at delivery, and have at least one atopic parent.^30^ The University of Cincinnati Institutional Review Board approved this study, and all enrolled parents gave written informed consent before their own and their infants’ study participation.

Of 407 and 762 infants in the HOME Study and CCAAPS, respectively, we excluded twin pregnancies (n = 18 for HOME; n = 32 for CCAAPS), children born <37 weeks gestation (HOME, n = 37; CCAAPS, n = 33), those missing exposure information (HOME, n = 17), covariates (HOME, n = 2; CCAAPS, n = 75), and those missing birthweight or gestational age (CCAAPS, n = 32). For our main analyses, we excluded preterm infants (<37 weeks) to reduce the effect of gestational age on birthweight and facilitate comparison between the two cohorts and with previous studies. The sample size for the analysis of birthweight was n = 333 for the HOME Study and n = 590 for CCAAPS. A total of n = 198 (HOME) and n = 459 (CCAAPS) children returned for the age 7- to 8-year follow-up and had anthropometry data available for our analysis of childhood adiposity (see Figure, Supplemental Digital Content 1; http://links.lww.com/EE/A55, flow diagram of participants included in each cohort and analysis). Due to differences in recruitment periods between the HOME Study and CCAAPS, there is no overlap in the participants enrolled in both cohorts. We were not able to assess if siblings were enrolled in both studies.

### Exposure assessment

We used a previously validated land use regression (LUR) model (*R*^2^ = 0.75) to estimate ECAT concentrations at each participant’s baseline address (at ≈20 weeks gestation for HOME and ≈6 months of age for CCAAPS).^30^ Briefly, between 2001 and 2006, research staff conducted ambient air sampling on a rotating basis across 24 sampling sites in the greater Cincinnati, OH region (see Figure, Supplemental Digital Content 2; http://links.lww.com/EE/A55, with the geographical overlap in cohorts and air sampling locations).^30^ First, we quantified the concentration of particulate matter (PM_2.5_). Next, we analyzed the filters using x-ray fluorescence to determine the concentration of 39 elements and thermal–optical transmittance to quantify elemental and organic carbon concentrations. We used a multivariate receptor model, UNMIX, to determine traffic sources contributing to the total PM_2.5_ concentrations. We identified a diesel-specific traffic signature using elemental source profiles from separate samples obtained at cluster sources and used this signature to quantify the contribution of diesel traffic to the PM_2.5_ concentrations collected at each sampling site.^32^ For each sampling site, we calculated the concentration of elemental carbon generated from traffic-related diesel combustion (μg/m^3^) and constructed the land use regression model.^30,32,33^ Because the land use regression model was developed using the arithmetic mean of all 24-hour samples collected at each site, the ECAT concentrations were estimates of long-term exposure and did not incorporate temporal variations in exposure.^30^ We used the ECAT concentration at the residential address as an estimate of children’s early-life exposure to traffic-related air pollution.

### Infant and child anthropometry

We abstracted the birthweight and gestational age of children from birth records in the HOME Study and relied on maternal report in CCAAPS. Trained research assistants measured weight and height when children were approximately 7–8 years of age in the HOME Study (range: 7.5–10 years) and CCAAPS (range: 6.4–7.9 years), respectively. Using World Health Organization (WHO) reference data (WHO, 2017), we calculated age- and sex-specific body mass index (BMI) z-scores.^34^

### Covariates

We collected sociodemographic and prenatal variables that may be related to both early-life ECAT concentrations and fetal or childhood growth. We identified potential confounders of the relationship of ECAT concentrations with fetal growth or child anthropometry a priori based on the existing literature and constructed directed acyclic graphs to visualize confounding relationships (see Figure, Supplemental Digital Content 3 and Supplemental Digital Content 4; http://links.lww.com/EE/A55, which illustrates variables confounding the association of ECAT with birthweight and childhood BMI).^35,36^ We assessed sociodemographic characteristics using standardized interviews administered at baseline; these included maternal race, marital status, age at delivery, education, and household income. The HOME Study assessed marital status at birth, whereas CCAAPS assessed marital status during late childhood.

We measured perinatal factors by medical chart review (HOME) or self-report (CCAAPS). These included parity, maternal tobacco smoke exposure, maternal prepregnancy BMI, and weight gain during pregnancy. Prenatal smoking status was assessed by self-report (HOME and CCAAPS) and confirmed via serum cotinine concentrations in the HOME Study. The HOME Study collected parity, whereas CCAAPS reported the number of children living at home under 18 years of age, which we used as a proxy for parity. The HOME Study abstracted maternal prepregnancy weight from medical charts, and we used this to calculate prepregnancy BMI. CCAAPS did not collect information about maternal weight pre- or postpregnancy. Thus, we were not able to adjust for prepregnancy BMI in the CCAAPS or pooled analyses. In CCAAPS, women self-reported the amount of weight gained during pregnancy, and in HOME, we abstracted this information from medical chart reviews.

### Statistical analysis

We used multivariable linear regression to evaluate the association of ECAT concentrations with (1) birthweight or (2) child BMI z-score at age 7–8 years in each cohort separately and in the pooled sample. In regression models, we adjusted for maternal race, household income, maternal age at delivery, maternal education, parity, maternal exposure to tobacco, weight gain during pregnancy, and maternal prepregnancy BMI. In the multivariable linear regression with birthweight as the outcome, we also adjusted for child sex. In each cohort, we considered ECAT as both a continuous and categorical variable (terciles). We used restricted cubic splines to assess the linearity of the dose-response relationship of ECAT and birthweight or BMI z-scores. We did not adjust for birthweight in the analyses of BMI z-score at age 7–8 years because birthweight is on the causal pathway between ECAT exposure and childhood BMI.

Before pooling the cohorts, we assessed whether the association of ECAT concentration with birthweight or BMI differed between cohorts. First, we assessed cohort heterogeneity by including terms for continuous ECAT concentrations, cohort, and their interaction term in adjusted models. Next, we compared multivariable models adjusting for cohort as a fixed effect to models adjusting for a random cohort effect (results not shown). For multivariable linear regression models in the pooled sample, we adjusted for cohort and covariates that were available in both cohorts.

We assessed the robustness of our models by comparing effect estimates from our final adjusted models with those from regression models not adjusting for maternal weight gain or prepregnancy BMI. We also conducted a sensitivity analysis including HOME Study infants born <37 weeks gestation in our analysis and adjusting for gestational age in the regression model. We were not able to conduct this sensitivity analysis in CCAAPS because infants born before 35 weeks were not enrolled in the study.

In addition, we conducted secondary analyses examining whether associations of ECAT with birthweight or childhood BMI varied by maternal race (non-Hispanic white or non-Hispanic black and other races), child sex, or household income (categorized as <$40,000 and ≥$40,000) in the individual cohorts and pooled sample. We first stratified participants based on maternal race, child sex, or household income and used adjusted linear regression models to estimate the relationship of ECAT with birthweight or BMI in each strata. Second, we assessed modification of the association between ECAT and each outcome by including a product interaction term between ECAT and the modifier of interest in a fully adjusted model. All analyses were performed using SAS v.9.4 (SAS Institutes, Inc., Cary, NC) and R version 3.4.0 (R Foundation for Statistical Computing, Vienna, Austria).

## Results

At baseline, the 923 participants included in this analysis across both cohorts were predominately non-Hispanic white (73%), age 25–35 years at time of delivery (59%), and nonsmokers (89%), with a household income >$70,000 (37%), bachelor’s degree or higher (51%), and previous child (61%) (Table 1). Compared with the HOME Study, a greater proportion of CCAAPS participants were non-Hispanic white, multiparous, and gained more weight during pregnancy. Excluded HOME Study participants born <37 weeks gestation were less likely to be non-Hispanic white compared with HOME Study participants included in our analysis (38% compared with 65%). Furthermore, excluded participants (n = 37) had a lower median income than HOME Study participants included in the analysis (median = $40,000; 25th, 75th percentile = $17,500, $75,000 vs. median = $55,000; 25th, 75th percentile = $22,500, $85,000), but had similar residential ECAT concentrations. There was not a difference in birthweight or BMI z-scores at age 7–8 years between the HOME Study and CCAAPS (see Table, Supplemental Digital Content 5; http://links.lww.com/EE/A55, which contains birthweight and BMI z-score summary statistics for both cohorts). Overall, the mean birthweight in the pooled sample was 3,487 g (standard deviation (SD) = 479) and the mean BMI z-score was 0.56 (SD = 1.29). The baseline characteristics of women who completed the follow-up visit when children were age 7–8 years old were similar to the full cohorts (Table 1).

**Table 1:**
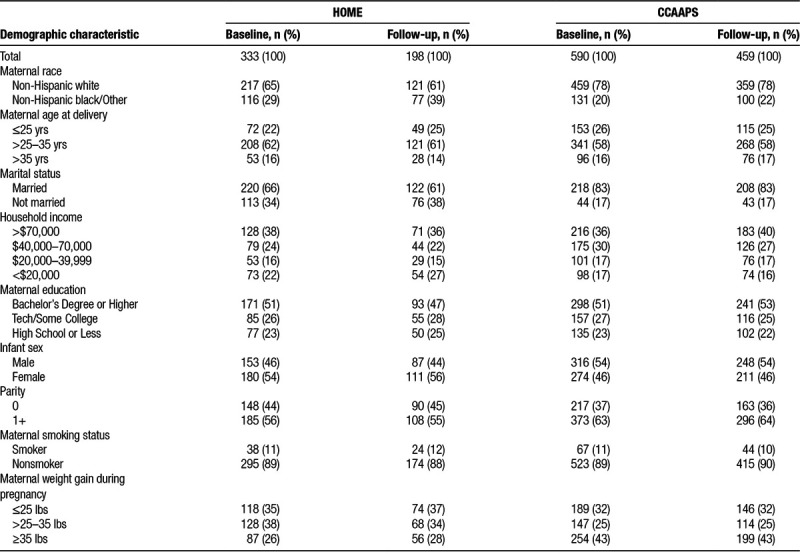
Maternal characteristics at baseline and follow-up at age 7–8 years among the HOME Study and CCAAPS participants

**Figure 1.**
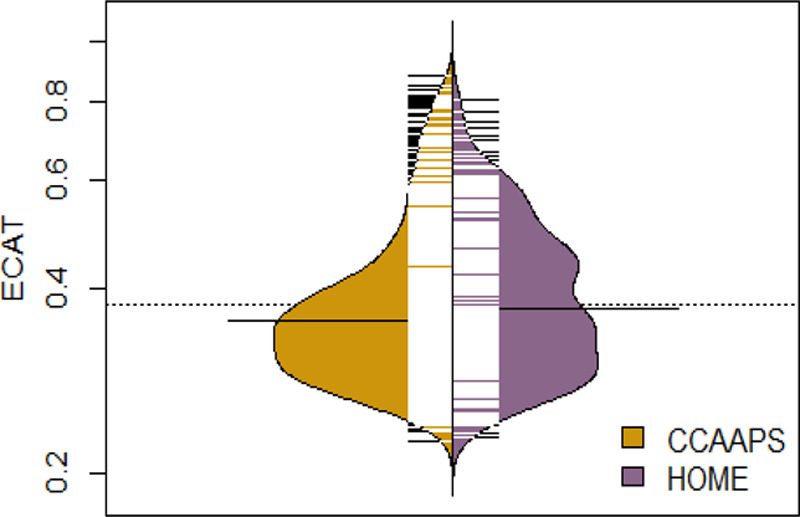
Bean plot of the distribution of elemental carbon attributable to traffic (ECAT) concentrations among the HOME Study and CCAAPS. ECAT concentration (μg/m3) is on a natural-log *y* axis. Each black and white mark represents a study participant, and the bold black lines near the center indicate median concentrations in each cohort.

ECAT concentrations estimated at residential addresses were comparable across the two cohorts (HOME median = 0.37 μg/m^3^; 25th, 75th percentile = 0.30, 0.46 μg/m^3^ vs. CCAAPS median = 0.35 μg/m^3^; 25th, 75th percentile=0.30, 0.42 μg/m^3^; Figure 1). Pooled ECAT concentrations had an interquartile range (IQR) of 0.15 μg/m^3^ (pooled 25th, 75th percentile: 0.30, 0.45). Across both cohorts, median ECAT concentrations were higher among women who were unmarried; non-Hispanic black or other race; younger than 25 years of age; had an annual household income <$20,000; had a high school diploma, high school equivalency diploma, or less; or smoked tobacco during pregnancy (see Table, Supplemental Digital Content 6; http://links.lww.com/EE/A55, with descriptive statistics for ECAT concentrations).

In both the individual cohort and pooled analyses, linearity tests using restricted cubic splines suggested that ECAT concentrations had a linear relationship with both birthweight and BMI z-scores (results not shown, all nonlinearity *P* values >0.45).

In adjusted models, residential ECAT concentrations were not associated with birthweight in the HOME Study (difference per IQR increase in ECAT: 34 g; 95% confidence interval (CI) = −28, 95), CCAAPS (difference per IQR increase in ECAT: 20 g; 95% CI = −23, 62), or the pooled sample (difference in birthweight: 30 g; 95% CI = −6, 66). Nor did we find evidence of an association between residential ECAT concentrations and BMI z-score at age 7–8 years in the HOME Study (difference per IQR increase in ECAT: −0.07; 95% CI = −0.27, 0.13), CCAAPS (difference per IQR increase in ECAT: −0.02; 95% CI = −0.16, 0.12), or the pooled sample (difference per IQR increase in ECAT: −0.04; 95% CI = −0.15, 0.08). Similarly, we found no association between residential ECAT concentrations and birthweight or BMI z-scores when we categorized ECAT concentrations into terciles (Tables 2 and 3). Results from models adjusting for cohort as a fixed effect were similar to those adjusting for a random cohort effect (results not shown). Furthermore, cohort did not modify the association between ECAT and birthweight or BMI (cohort and ECAT interaction term *P* values = 0.83 and 0.28, for birthweight and BMI z-score analyses, respectively).

**Table 2:**
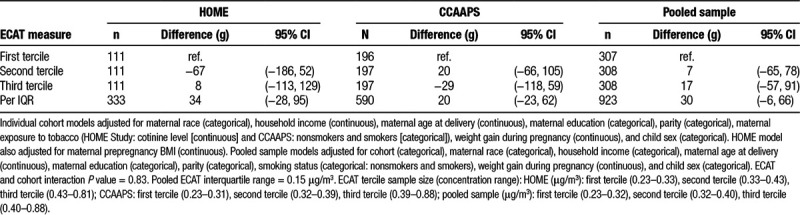
Adjusted difference in term birthweight across ECAT concentration terciles and per interquartile range increase in ECAT concentration (HOME Study and CCAAPS)

**Table 3:**
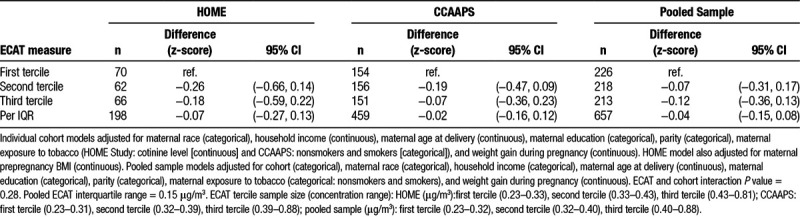
Adjusted difference in body mass index z-scores at age 7–8 years across ECAT concentration terciles and per interquartile range change in ECAT concentration (HOME Study and CCAAPS)

In sensitivity analyses, the association of ECAT concentration with birthweight or BMI was similar in fully adjusted models and models not adjusted for pregnancy weight gain or maternal prepregnancy BMI (see Tables, Supplemental Digital Content 7; http://links.lww.com/EE/A55 and Supplemental Digital Content 8; http://links.lww.com/EE/A55, characterizing the association in sensitivity analyses). Furthermore, inclusion of infants born <37 weeks gestation in the HOME Study and adjustment for gestational age did not meaningfully alter the association between ECAT concentration and birthweight in adjusted models. In the individual cohort analyses, the association of ECAT concentration with birthweight or BMI z-score did not meaningfully vary across strata of maternal race, child sex, or household income (all interaction *P* values >0.12; see Tables, Supplemental Digital Content 9; http://links.lww.com/EE/A55 and Supplemental Digital Content 10; http://links.lww.com/EE/A55, which contain stratified analyses). In the pooled analysis, infant sex modified the association between ECAT concentrations and birthweight. Among male newborns, higher ECAT concentrations were associated with higher birthweight (61 g; 95% CI = 9, 113), but we observed no association among female newborns (−9 g; 95% CI = −58, 41; infant sex and ECAT interaction *P* value = 0.05).

## Discussion

Our results do not suggest an association of traffic-related air pollution exposure during early life with lower birthweight or higher BMI at age 7–8 years. Results from our secondary analyses suggest that infant sex could potentially modify the association between residential traffic-related air pollution exposure and birthweight; however, these results warrant further investigation. We did not find that the relationship between ECAT and BMI z-scores varied by maternal race, child sex, or household income.

Exhaust generated from diesel combustion consists of fine (0.1–2.5 μm) and ultrafine particles (<0.1 μm) which can adversely affect health and development by penetrating deep into the lungs, entering the bloodstream, and causing oxidative stress.^37–39^ The fetus may be particularly vulnerable to environmental pollutants, like diesel exhaust, because of immature detoxification pathways and increased sensitivity to environmental stressors.^40^ Prenatal traffic-related air pollution exposure could impact childhood adiposity by altering metabolic function during fetal development. Previous studies have reported early-life traffic-related air pollution exposure is associated with an increase in leptin and adiponectin concentrations in cord blood,.^27,41,42^

Leptin and adiponectin are adipocytokines hypothesized to be related to fetal, infant, and child growth, as well as obesity risk. In a larger sample, Bell et al^43^ (2010) reported on average a 6 g (95% CI = −11, 0) lower birthweight per IQR increase (1.1 μg/m^3^) in gestational exposure to ambient elemental carbon. Similarly, Kingsley et al^19^ (2017) and Bell et al^15^ (2007), among others, report an association between model-based and monitor-based PM_2.5_ exposure estimates and birthweight (12.1 g lower birthweight per IQR [2.5 μg/m^3^], 95% CI = −24.2, −0.1; 14.7 g lower birthweight per IQR [2.2 μg/m^3^], 95% CI = −17.1, −12.3, respectively).^14,43^ Several studies have also consistently reported an association between traffic density and estimated exposure to traffic-related air pollutants, including PM_2.5_, elemental carbon, and nitrogen oxides, and higher odds of term low birthweight.^17,18,44^

Our findings related to the association between traffic-related air pollution and birthweight may be impacted by our modest sample size and lack of variation in exposure estimates. Although the distribution of birthweight measurements in our analysis is comparable to previous studies, the ECAT concentrations are significantly lower and less variable than estimates of total elemental carbon concentrations from traffic sources in general. For example, our ECAT estimates only represent a portion of total elemental carbon. The mean ECAT concentration for the combined cohorts was 0.39 μg/m^3^ (SD = 0.12), whereas the mean of the total elemental carbon concentrations from motor vehicles in the study by Bell et al^43^ (2010) was 1.04 μg/m^3^ (SD = 0.60). Our study adds to this existing literature by focusing on lower exposure concentrations from a more specific traffic type.

Previous studies have also found an association of traffic-related air pollution with childhood obesity; however, results have varied based on exposure assessment method and timing. For example, similar to our results, both Fleisch et al^45^ (2017) and Fioravanti et al^46^ (2018) reported no association between exposure to estimated concentrations of traffic-related air pollutants, such as black carbon, particulate matter, or nitrogen oxides, and childhood obesity. However, Fleisch et al^45^ (2017) did report an association between proximity to roadway and greater fat mass around age 7 years. Although Kim et al^16^ (2018) also reported no association between gestational exposure to freeway nitrogen oxides and childhood obesity, they did report an association between early-life exposure and BMI at age 10 when adjusting for later childhood exposure, a period of time when traffic-related air pollution exposure may also be associated with higher childhood BMI.^9^ However, results from Kim et al^16^ (2018) only suggest an association between later childhood exposure to nonfreeway traffic and higher BMI at age 10, but not freeway traffic.

We speculate that differences in built environments surrounding distinct roadway types, such as greenspace and noise barriers, and access to recreational space in areas of dense roadways could influence exposure and childhood obesity risk; therefore, our findings could be influenced by residual confounding from our lack of consideration for these factors (see Figure, Supplemental Digital Content 11; http://links.lww.com/EE/A55, which illustrates potential confounders that were not included in the analyses). Additionally, we did not account for exposure during childhood, which could vary from gestational or early-life exposure. For example, previous analyses of the CCAAPS cohort report that 54% of participants moved at least once during childhood; changes in residential address could impact longitudinal exposure to traffic-related air pollution.^47^ Future studies with spatio-temporal exposure models should consider including traffic exposure during childhood given that it could be an additional period of susceptibility.

Previous evidence suggests that maternal race, child sex, or household income could modify the association of air pollution exposure with fetal growth or childhood adiposity.^15,44,48,49^ Although the research is limited, prior studies suggest that the associations of particulate matter with lower birthweight and higher childhood BMI are stronger in males.^10,49,50^ Additionally, males may be more susceptible to preterm birth associated with exposure to some air pollutants.^51^ Our results suggesting that higher birthweight may be associated with ECAT exposure among males warrant further investigation. Future studies with larger sample sizes and other fetal growth measures are needed to better characterize sex-specific associations between traffic-related air pollution exposure and fetal growth.

There are some limitations to our present study. First, limiting our main analyses to term infants born at 37 weeks gestation or after may result in selection bias. Previous studies have reported a relationship of traffic-related pollution exposure with preterm birth.^52,53^ If traffic-related air pollution exposure is associated with preterm birth in the Cincinnati, OH, region, then infants with higher ECAT exposure would be excluded from our analyses, which we expect to bias our results toward the null. Our sensitivity analysis which included HOME Study participants born preterm (i.e., <37 weeks) does not suggest that the exclusion of preterm infants in the main analyses affected our results. However, the number of infants born preterm in the HOME Study is small, and we were not able to examine associations among preterm infants in CCAAPS because infants born before 35 weeks were not enrolled in the study. Second, CCAAPS inclusion criteria also required infants to have at least one atopic parent. This criterion, however, does not appear to bias our findings, given that the two cohorts yielded similar results. Third, we assessed childhood obesity using body mass index which may not accurately reflect body composition. Fourth, we did not consider potential confounding due to maternal or child exercise and diet. Diet and exercise impact childhood adiposity and can be associated with traffic-related air pollution exposure through socioeconomic status. Moreover, we did not consider exposure to traffic noise or greenspace, which could have resulted in some residual confounding. Finally, we estimated early-life ECAT exposure based on one residential address, although women may have moved. Moreover, we used the land use regression model to estimate long-term ECAT exposure; therefore, we were unable to differentiate effects of ECAT exposure during specific time periods and did not consider ECAT exposure during childhood. Housing characteristics and individual time-activity patterns could also contribute to misclassification of exposure, which on average is expected to bias our results toward the null.

On the other hand, our analysis also had several strengths. Body mass index was calculated using anthropometric measurements taken by trained assistants and standardized protocols. In addition, we were able to compare our findings across the two cohorts which recruited participants from overlapping geographical areas during the same time period and assessed similar covariates. Lastly, we used a validated land use regression model that has been used in other health studies to estimate exposure at women’s residential addresses.^30,33^

## Conclusions

In contrast to previous studies and our hypothesis, we did not find that traffic-related air pollution exposure was associated with lower birthweight or higher childhood adiposity at age 7–8 years in these two cohorts or the pooled sampled. Our results should be cautiously interpreted given prior research and the limitations of our study, including the modest sample size, relatively low ECAT concentrations, and our ability to examine ECAT only during the prenatal period. Future studies with larger sample sizes and temporally resolved traffic-related air pollution exposure models for intraurban areas may help clarify inconsistent research findings. Given the lifelong impact of low birthweight and childhood obesity, identifying specific sources of traffic pollution that increase obesity risk and built environment characteristics that can protect against exposure should be of great public health interest.

## Conflicts of interest statement

B.P.L. reported serving as an expert witness in childhood lead poisoning cases, for which he has not received any compensation. J.M.B. was financially compensated for serving as an expert witness for plaintiffs in litigation related to tobacco smoke exposures. The other authors have no conflicts to report.

This work was supported by grants R01 ES024381, R01 ES020349, R01 ES011170, R21 ES023073, and P42 ES013660 from the US National Institute of Environmental Health Sciences (NIEHS) and National Institutes of Health (NIH). C.G.S. is partially supported by the Institute at Brown for Environment and Society. The contents of this report are solely the responsibility of the authors and do not necessarily represent the official views of the sponsoring organizations.

Interested investigators should contact J.M.B. (joseph_braun_1@brown.edu) and Dr. Kimberly Yolton (kimberly.yolton@cchmc.org) to discuss collaborative opportunities, obtain additional information about the Health Outcomes and Measures of the Environment (HOME) Study, and request a project proposal form. The HOME Study Data Sharing Committee meets regularly to review proposed research projects and ensure that they do not overlap with extant projects and are an efficient use of scarce resources.

## ACKNOWLEDGMENTS

The authors would like to acknowledge Christopher Wolfe for assisting with the production of figures.

## Supplementary Material
