# Supplementary material for: Early-life exposure to traffic-related air pollution and child anthropometry
Source: Environ Epidemiol. 2019 Aug 29;3(5):e061. doi: 10.1097/EE9.0000000000000061 (PMC7461703; doi:10.1097/EE9.0000000000000061)
Supplement: Supplementary file 1 [file ee9-3-e061-s001.docx]

**Supplemental Digital Content for:**

Early-life exposure to traffic-related air pollution and child anthropometry

**Authors**: Clara G. Sears ^a^, Catrina Mueller-Leonhard ^a^, Gregory A. Wellenius ^a^, Aimin Chen ^b^, Patrick Ryan ^c, d^, Bruce P. Lanphear ^e^, and Joseph M. Braun ^a^

^a^ Department of Epidemiology, Brown University, Providence, RI, United States

^b^ Department of Environmental Health, University of Cincinnati College of Medicine, Cincinnati, OH, United States

^c^ Department of Pediatrics, University of Cincinnati, College of Medicine, Cincinnati, OH, United States

^d^ Division of Biostatistics and Epidemiology, Cincinnati Children’s Hospital Medical Center, Cincinnati, OH, United States

^e^ Faculty of Health Sciences, Simon Fraser University, Burnaby, BC, Canada

**List of Supplemental Digital Content:**

Supplemental Digital Content 1. Flow diagram of participants included in each cohort for the HOME Study and CCAAPS cohorts.

Supplemental Digital Content 2. Figure with map illustrating the geographical distribution of participants in HOME Study and CCAAPS.

Supplemental Digital Content 3. Figure with directed acyclic graph of variables confounding the association of ECAT with birthweight.

Supplemental Digital Content 4. Figure with directed acyclic graph of variables confounding the association of ECAT with childhood BMI.

Supplemental Digital Content 5: Summary statistics of term birthweight (grams) and BMI z-score for HOME Study and CCAAPS participants at enrollment.

Supplemental Digital Content 6. Table with median (25th and 75th percentile) maternal residential elemental carbon attributable to traffic (μg/m³) concentrations by covariate and cohort.

Supplemental Digital Content 7. Table with adjusted difference in term birthweight per interquartile range increase in maternal resident ECAT concentrations: Sensitivity analyses.

Supplemental Digital Content 8. Table with adjusted difference in BMI z-score at age 7-8 years per interquartile range change in maternal residential ECAT concentrations: Sensitivity analyses.

Supplemental Digital Content 9. Table with adjusted difference in term birthweight per interquartile range increase in residential ECAT concentrations stratified by maternal race, household income, and infant sex (HOME Study and CCAAPS).

Supplemental Digital Content 10. Table with adjusted difference in body mass index z-scores at age 7-8 years per interquartile range increase in residential ECAT concentration stratified by maternal race, household income and infant sex (HOME Study and CCAAPS).

Supplemental Digital Content 11. Directed acyclic graph of variables confounding the association of ECAT with birthweight and childhood BMI.

**Supplemental Digital Content 1.** Flow diagram of participants included in each cohort for the HOME Study and CCAAPS cohorts.

407 HOME Study Participants

55 Excluded

♦  18 twin pregnancies

♦  37 born <37 weeks

19 Missing data

♦  17 exposure data

♦  2 covariate data

333 included in birthweight analysis

198 included in childhood BMI analysis

762 CCAAPS

Participants

65 Excluded

♦  32 twin pregnancies

♦  33 born <37 weeks

107 Missing data

♦  32 outcome data

♦  75 covariate data

590 included in birthweight analysis

459 included in childhood BMI analysis

#### **Supplemental Digital Content 2. Geographical distribution of participants in HOME Study and CCAAPS.**


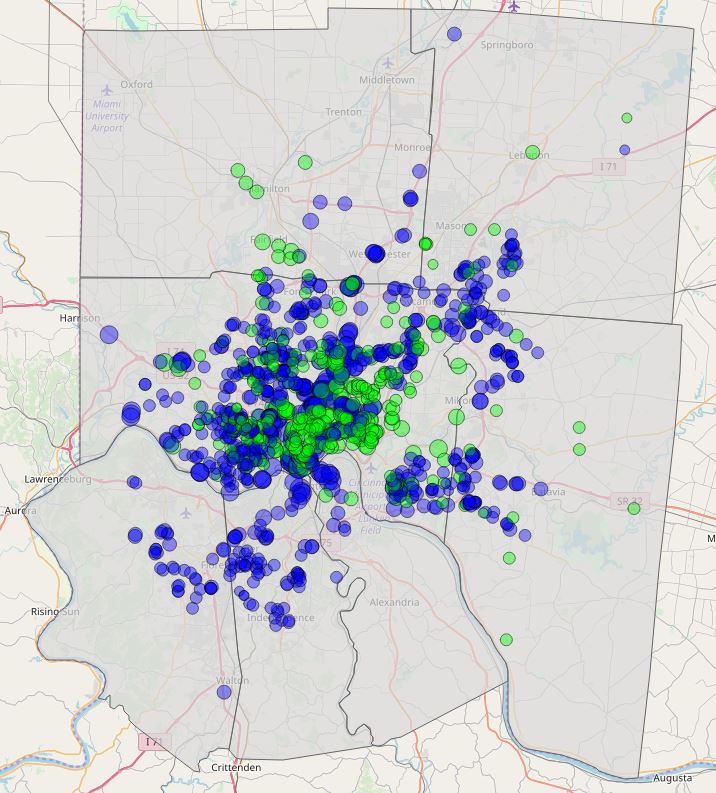


**Cohort**

HOME Study

CCAAPS


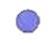

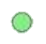


**
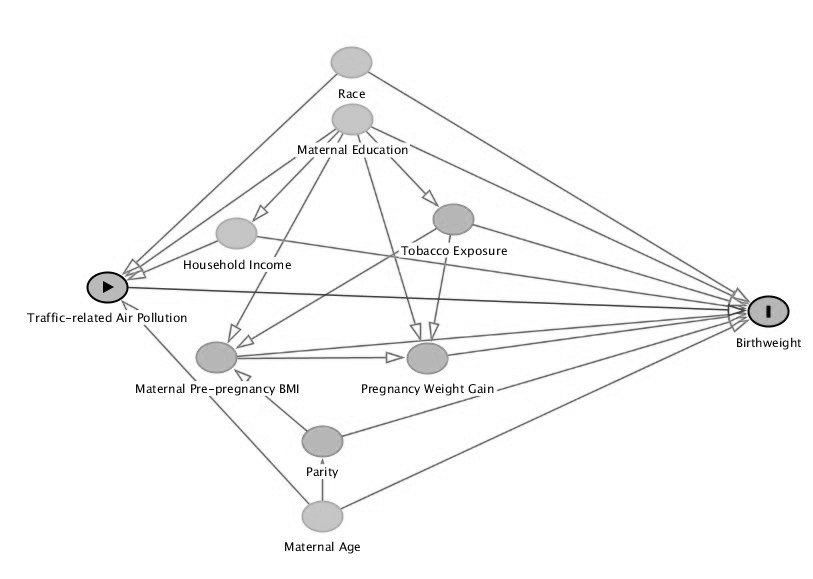
**

**Supplemental Digital Content 3. Directed acyclic graph of variables potentially confounding the association of ECAT with birthweight.**

**
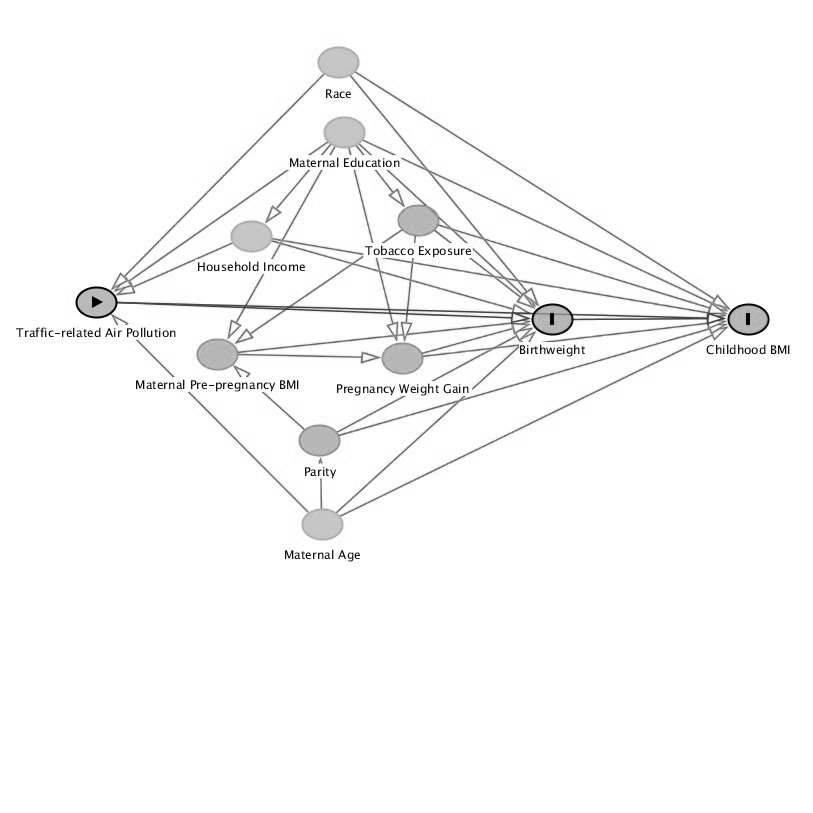
**

**Supplemental Digital Content 4. Directed acyclic graph of variables potentially confounding the association of ECAT with childhood BMI**

| Supplemental Digital Content 5: Summary statistics of term birthweight (grams) and BMI z-score for HOME Study and CCAAPS participants at enrollment. | | | | | | |
| --- | --- | --- | --- | --- | --- | --- |
|  | **HOME** | | | **CCAAPS** | | |
|  | Baseline  n (%) | Mean Birthweight  (SD) | Mean BMI  z-score  (SD) | Baseline  n (%) | Mean Birthweight  (SD) | Mean BMI  z-score  (SD) |
| Total | 333 (100) | 3469 (537) | 0.59 (1.27) | 590 (100) | 3497 (443) | 0.55 (1.29) |
| Maternal Race |  |  |  |  |  |  |
| Non-Hispanic White | 217 (65) | 3578 (552) | 0.37 (1.09) | 459 (78) | 3545 (437) | 0.43 (1.29) |
| Non-Hispanic Black | 97 (29) | 3235 (447) | 1.00 (1.37) | 121 (20) | 3322 (406) | 1.02 (1.23) |
| Other | 19 (6) | 3424 (390) | 0.37 (2.00) | 10 (2) | 3442 (623) | 0.48 (0.50) |
| Maternal Age at Delivery |  |  |  |  |  |  |
| $\boldsymbol{\leq}$ 25 years | 72 (22) | 3196 (439) | 0.69 (1.54) | 153 (26) | 3401 (452) | 0.82 (1.22) |
| $\boldsymbol{>}$ 25 - 35 years | 208 (62) | 3547 (525) | 0.59 (1.19) | 341 (58) | 3540 (431) | 0.44 (1.32) |
| $\boldsymbol{>}$ 35 years | 53 (16) | 3535 (591) | 0.39 (1.15) | 96 (16) | 3499 (452) | 0.53 (1.30) |
| Marital Status |  |  |  |  |  |  |
| Married | 220 (66) | 3572 (542) | 0.46 (1.16) | 218 (83) | 3553 (447) | 0.44 (1.24) |
| Not Married | 113 (34) | 3270 (469) | 0.79 (1.42) | 44 (17) | 3268 (389) | 1.02 (1.51) |
| Household Income |  |  |  |  |  |  |
| $\boldsymbol{>}$ $70,000 | 128 (38) | 3547 (520) | 0.49 (1.13) | 216 (36) | 3562 (430) | 0.27 (1.14) |
| $40,000 - 70,000 | 79 (24) | 3667 (573) | 0.33 (0.97) | 175 (30) | 3502 (447) | 0.54 (1.38) |
| $20,000 - 39,999 | 53 (16) | 3395 (493) | 0.62 (1.45) | 101 (17) | 3483 (481) | 0.68 (1.33) |
| $\boldsymbol{<}$ $20,000 | 73 (22) | 3174 (419) | 0.89 (1.52) | 98 (17) | 3361 (397) | 1.11 (1.29) |
| Maternal Education |  |  |  |  |  |  |
| >Bachelor’s Degree | 171 (51) | 3590 (552) | 0.52 (1.12) | 298 (51) | 3549 (440) | 0.31 (1.22) |
| Tech/ Some College | 85 (26) | 3377 (464) | 0.29 (1.38) | 157 (27) | 3465 (418) | 0.56 (1.36) |
| High School or Less | 77 (23) | 3303 (518) | 1.03 (1.34) | 135 (23) | 3420 (466) | 1.10 (1.24) |
| Infant Gender |  |  |  |  |  |  |
| Male | 153 (46) | 3609 (561) | 0.51 (1.29) | 316 (54) | 3523 (460) | 0.61 (1.39) |
| Female | 180 (54) | 3351 (486) | 0.64 (1.27) | 274 (46) | 3467 (422) | 0.48 (1.17) |
| Parity |  |  |  |  |  |  |
| 0 | 148 (44) | 3408 (558) | 0.45 ( 1.20) | 217 (37) | 3466 (445) | 0.56 (1.43) |
| 1+ | 185 (56) | 3519 (515) | 0.70 (1.33) | 373 (63) | 3515 (441) | 0.55 (1.22) |
| Maternal Smoking Status |  |  |  |  |  |  |
| Smoker | 38 (11) | 3304 (498) | 0.83 (1.57) | 67 (11) | 3371 (421) | 1.00 (1.39) |
| Non-Smoker | 295 (89) | 3491 (539) | 0.55 (1.23) | 523 (89) | 3513 (444) | 0.50 (1.28) |
| Maternal Weight Gain During Pregnancy |  |  |  |  |  |  |
| $\boldsymbol{\leq}$ 25 lbs. | 118 (35) | 3328 (532) | 0.52 (1.24) | 189 (32) | 3398 (433) | 0.73 (1.40) |
| $\boldsymbol{>}$ 25 - 35 lbs. | 128 (38) | 3465 (514) | 0.50 (1.26) | 147 (25) | 3440 (443) | 0.41 (1.34) |
| $\boldsymbol{\geq}$ 35 lbs. | 87 (26) | 3667 (520) | 0.78 (1.34) | 254 (43) | 3604 (429) | 0.50 (1.17) |
| Pooled Sample- mean birthweight = 3487g (SD = 479) and the mean BMI z-score = 0.56 (SD=1.29)  Total sample size for BMI analysis at age 7/8 years: HOME Study = 198; CCAAPS = 459  Abbreviations: HOME, Health Outcomes and Measures of the Environment; CCAAPS, Cincinnati Childhood and Air Pollution Study; BMI, body mass index. | | | | | | |

| Supplemental Digital Content 6. Median (25^th^ and 75^th^ percentile) maternal residential elemental carbon attributable to traffic (μg/m³) concentrations by covariate and cohort (HOME Study and CCAAPS). | | | |
| --- | --- | --- | --- |
|  | HOME | CCAAPS |  |
|  | ECAT median  (25%, 75%) | ECAT median  (25%, 75%) |  |
| Overall | 0.37 (0.30, 0.46) | 0.35 (0.30, 0.42) |  |
| Maternal Race |  |  |  |
| Non-Hispanic White | 0.35 (0.29, 0.45) | 0.34 (0.30, 0.40) |  |
| Non-Hispanic Black | 0.43 (0.34, 0.50) | 0.39 (0.33, 0.48) |  |
| Other | 0.37 (0.31, 0.47) | 0.39 (0.32, 0.51) |  |
| Maternal Age at Delivery |  |  |  |
| $\boldsymbol{\leq}$ 25 years | 0.41 (0.33, 0.49) | 0.39 (0.32, 0.47) |  |
| $\boldsymbol{>}$ 25 – 35 years | 0.35 (0.30, 0.45) | 0.34 (0.30, 0.40) |  |
| $\boldsymbol{>}$ 35 years | 0.40 (0.33, 0.49) | 0.34 (0.29, 0.39) |  |
| Marital Status |  |  |  |
| Married | 0.36 (0.30, 0.44) | 0.34 (0.29, 0.40) |  |
| Not Married | 0.42 (0.32, 0.50) | 0.43 (0.32, 0.55) |  |
| Household Income |  |  |  |
| $\boldsymbol{>}$ $70,000 | 0.37 (0.32, 0.46) | 0.34 (0.29, 0.38) |  |
| $40,000 - 70,000 | 0.32 (0.28, 0.40) | 0.33 (0.29, 0.40) |  |
| $20,000 - 39,999 | 0.34 (0.29, 0.47) | 0.36 (0.31, 0.46) |  |
| $\boldsymbol{<}$ $20,000 | 0.43 (0.35, 0.55) | 0.41 (0.35, 0.50) |  |
| Maternal Education |  |  |  |
| Bachelor’s Degree or Higher | 0.36 (0.30, 0.45) | 0.34 (0.29, 0.39) |  |
| Tech/ Some College | 0.36 (0.30, 0.47) | 0.35 (0.30, 0.44) |  |
| High School or Less | 0.39 (0.31, 0.50) | 0.40 (0.33, 0.50) |  |
| Infant Gender |  |  |  |
| Male | 0.37 (0.30, 0.48) | 0.35 (0.30, 0.42) |  |
| Female | 0.36 (0.31, 0.45) | 0.35 (0.30, 0.42) |  |
| Parity |  |  |  |
| 0 | 0.37 (0.31, 0.46) | 0.35 (0.30, 0.43) |  |
| 1+ | 0.37 (0.30, 0.46) | 0.35 (0.30, 0.42) |  |
| Maternal Smoking Status |  |  |  |
| Smoker | 0.43 (0.31, 0.53) | 0.39 (0.31, 0.52) |  |
| Non-Smoker | 0.36 (0.30, 0.46) | 0.35 (0.30, 0.41) |  |
| Maternal Weight Gain During Pregnancy |  |  |  |
| $\boldsymbol{\leq}$ 25 lbs. | 0.35 (0.30, 0.48) | 0.36 (0.30, 0.42) |  |
| $\boldsymbol{>}$ 25 - 35 lbs. | 0.37 (0.31, 0.45) | 0.34 (0.30, 0.39) |  |
| $\boldsymbol{\geq}$ 35 lbs. | 0.39 (0.31, 0.47) | 0.35 (0.30, 0.45) |  |
| Maternal BMI at baseline |  |  |  |
| $\boldsymbol{<}$ 25 | 0.38 (0.32, 0.46) | - (-) |  |
| 25 - $\boldsymbol{<}$ 30 | 0.35 (0.29, 0.43) | - (-) |  |
| $\boldsymbol{\geq}$ 30 | 0.38 (0.31, 0.49) | - (-) |  |
| Abbreviations: ECAT, Elemental carbon attributable to traffic; HOME, Health Outcomes and Measures of the Environment; CCAAPS, Cincinnati Childhood and Air Pollution Study. | | |  |

| **Supplemental Digital Content 7. Adjusted difference in term birthweight per interquartile range increase in maternal resident ECAT concentrations: Sensitivity analyses (HOME Study and CCAAPS)** | | | | | | |
| --- | --- | --- | --- | --- | --- | --- |
|  | **HOME** | | **CCAAPS** | | **Pooled Sample** | |
| **Model** | Difference  (grams) | 95% CI | Difference (grams) | 95% CI | Difference (grams) | 95% CI |
| Model 1: Fully adjusted | 34 | (-28, 95) | 20 | (-23, 62) | 30 | (-6, 66) |
| Model 2: Unadjusted | -17 | (-90, 54) | -6 | (-48, 36 ) | -10 | (-47, 27) |
| Model 3: Not adjusted for maternal weight | 21 | (-48, 91) | 20 | (-22, 63) | 30 | (-7, 67) |
| Model 4: Preterm infants included | 38 | (-20, 97) | - | - | - | - |
| Model 1: Individual cohort models adjusted for maternal race (categorical), household income (continuous), maternal age at delivery (continuous), maternal education (categorical), parity (categorical), maternal exposure to tobacco (HOME Study: cotinine level (continuous) and CCAAPS: Non-smokers and Smokers (categorical), weight gain during pregnancy (continuous), and child sex (categorical). HOME model also adjusted for maternal pre-pregnancy BMI (continuous). Pooled sample models adjusted for cohort (categorical), maternal race (categorical), household income (categorical), maternal age at delivery (continuous), maternal education (categorical), parity (categorical), smoking status (categorical: non-smokers and smokers), weight gain during pregnancy (continuous), and child sex (categorical).  Model 2: Pooled model adjusted for cohort (categorical).  Model 3: Adjusted for maternal race (categorical), household income (continuous), maternal age at delivery (continuous), maternal education (categorical), parity (categorical), maternal exposure to tobacco (HOME Study: cotinine level (continuous); CCAAPS: Non-smokers and Smokers (categorical); Pooled Sample: Non-smokers and Smokers (categorical)), and infant sex (categorical). Pooled sample models adjusted for cohort (categorical), maternal race (categorical), household income (categorical), maternal age at delivery (continuous), maternal education (categorical), parity (categorical), smoking status (categorical: non-smokers and smokers), and child sex (categorical).  Model 4 Adjusted for maternal race (categorical), household income (continuous), maternal age at delivery (continuous), maternal education (categorical), parity (categorical), maternal exposure to tobacco (HOME Study: cotinine level (continuous) and CCAAPS: Non-smokers and Smokers (categorical), weight gain during pregnancy (continuous), child sex (categorical), maternal pre-pregnancy BMI (continuous), and gestational age (continuous).  Abbreviations: ECAT, Elemental carbon attributable to traffic; HOME, Health Outcomes and Measures of the Environment; CCAAPS, Cincinnati Childhood and Air Pollution Study. | | | | | | |

| **Supplemental Digital Content 8. Adjusted difference in BMI z-score at age 7-8 years per interquartile range change in maternal residential ECAT concentrations: Sensitivity analyses (HOME Study and CCAAPS)** | | | | | | | |
| --- | --- | --- | --- | --- | --- | --- | --- |
|  | **HOME** | | **CCAAPS** | | **Pooled Sample** | |  |
|  | Difference  (z-score) | 95% CI | Difference (z-score) | 95% CI | Difference (z-score) | 95% CI |  |
| Model 1: Fully adjusted | -0.07 | (-0.27, 0.13) | -0.02 | (-0.16, 0.12) | -0.04 | (-0.15, 0.08) |  |
| Model 2: Unadjusted | -0.01 | (-0.22, 0.19) | 0.09 | (-0.04,0.23) | 0.06 | (-0.05, 17) |  |
| Model 3: Not adjusted for maternal weight | -0.11 | (-0.32, 0.10) | -0.02 | (-0.16, 0.12) | -0.03 | (-0.15, 0.08) |  |
| Model 1: Individual cohort models adjusted for maternal race (categorical), household income (continuous), maternal age at delivery (continuous), maternal education (categorical), parity (categorical), maternal exposure to tobacco (HOME Study: cotinine level (continuous) and CCAAPS: Non-smokers and Smokers (categorical), and weight gain during pregnancy (continuous). HOME model also adjusted for maternal pre-pregnancy BMI (continuous).  Pooled sample models adjusted for cohort (categorical), maternal race (categorical), household income (categorical), maternal age at delivery (continuous), maternal education (categorical), parity (categorical), maternal exposure to tobacco (categorical: non-smokers and smokers), and weight gain during pregnancy (continuous).  Model 2: Pooled model adjusted for cohort (categorical).  Model 3: Adjusted for maternal race (categorical), household income (continuous), maternal age at delivery (continuous), maternal education (categorical), parity (categorical), maternal exposure to tobacco (HOME Study: cotinine level (continuous); CCAAPS: Non-smokers and Smokers (categorical); Pooled Sample: Non-smokers and Smokers (categorical)). Pooled sample models adjusted for cohort (categorical), maternal race (categorical), household income (categorical), maternal age at delivery (continuous), maternal education (categorical), parity (categorical), and maternal exposure to tobacco (categorical: non-smokers and smokers).  Abbreviations: ECAT, Elemental carbon attributable to traffic; HOME, Health Outcomes and Measures of the Environment; CCAAPS, Cincinnati Childhood and Air Pollution Study. | | | | | | |  |

| **Supplemental Digital Content 9. Adjusted difference in term birthweight per interquartile range increase in residential ECAT concentrations stratified by maternal race, household income, and infant sex (HOME Study and CCAAPS)** | | | | | | | | | | | |
| --- | --- | --- | --- | --- | --- | --- | --- | --- | --- | --- | --- |
|  | | **HOME** | | | **CCAAPS** | | | **Pooled Sample** | | | |
|  | Difference  (grams) | | 95% CI | Interaction p-value | Difference (grams) | 95% CI | Interaction p-value | | Difference (grams) | 95% CI | Interaction p-value |
| **Maternal Race** |  | |  | 0.29 |  |  | 0.40 | |  |  | 0.15 |
| Non-Hispanic White | 66 | | (-26, 159) |  | 22 | (-28, 72) |  | | 43 | (-2, 88) |  |
| Non-Hispanic Black | -12 | | (-93, 69) |  | -16 | (-99, 67) |  | | -18 | (-78, 43) |  |
| **Household Income** |  | |  | 0.12 |  |  | 0.51 | |  |  | 0.45 |
| $<$ $40,000 | -7 | | (-88, 74) |  | 34 | (-26, 94) |  | | 12 | (-38, 62) |  |
| $\geq$$40,000 | 103 | | (6, 200) |  | 1 | (-58, 61) |  | | 34 | (-17, 85) |  |
| **Infant Sex** |  | |  | 0.51 |  |  | 0.13 | |  |  | 0.05 |
| Female | 0 | | (-81, 81) |  | -20 | (-81, 40) |  | | -9 | (-58, 41) |  |
| Male | 44 | | (-49, 138) |  | 52 | (-7, 112) |  | | 61 | (9, 113) |  |
| Individual cohort models also adjusted for maternal age at delivery (continuous), maternal education (categorical), parity (categorical), maternal exposure to tobacco (HOME Study: cotinine level (continuous) and CCAAPS: Non-smokers and Smokers (categorical), weight gain during pregnancy (continuous), and child sex (categorical). HOME model also adjusted for maternal pre-pregnancy BMI (continuous). Pooled sample models adjusted for cohort (categorical), maternal age at delivery (continuous), maternal education (categorical), parity (categorical), smoking status (categorical: non-smokers and smokers), weight gain during pregnancy (continuous), and child sex (categorical).  Abbreviations: ECAT, Elemental carbon attributable to traffic; HOME, Health Outcomes and Measures of the Environment; CCAAPS, Cincinnati Childhood and Air Pollution Study. | | | | | | | | | | | |

| **Supplemental Digital Content 10. Adjusted difference in body mass index z-scores at age 7-8 years per interquartile range increase in residential ECAT concentration stratified by maternal race, household income and infant sex (HOME Study and CCAAPS)** | | | | | | | | | |
| --- | --- | --- | --- | --- | --- | --- | --- | --- | --- |
|  | **HOME** | | | **CCAAPS** | | | **Pooled Sample** | | |
|  | Difference  (z-score) | 95% CI | Interaction p-value | Difference  (z-score) | 95% CI | Interaction p-value | Difference  (z-score) | 95% CI | Interaction p-value |
| **Maternal Race** |  |  | 0.97 |  |  | 0.17 |  |  | 0.59 |
| Non-Hispanic White | -0.10 | (-0.36, 0.18) |  | -0.10 | (-0.26, 0.07) |  | -0.07 | (-0.22, 0.07) |  |
| Non-Hispanic Black | -0.13 | (-0.48, 0.21) |  | 0.14 | (-0.11, 0.39) |  | 0.02 | (-0.19, 0.23) |  |
| **Household Income** |  |  | 0.48 |  |  | 0.20 |  |  | 0.84 |
| $<$ $40,000 | -0.15 | (-0.46, 0.17) |  | 0.08 | (-0.12, 0.28) |  | -0.03 | (-0.20, 0.14) |  |
| $\geq$ $40,000 | 0.08 | (-0.21, 0.36) |  | -0.09 | (-0.28, 0.10) |  | -0.04 | (-0.20, 0.12) |  |
| **Infant Sex** |  |  | 0.85 |  |  | 0.38 |  |  | 0.67 |
| Female | -0.12 | (-0.39, 0.14) |  | 0.07 | (-0.13, 0.26) |  | -0.02 | (-0.18, 0.15) |  |
| Male | -0.06 | (-0.35, 0.22) |  | -0.08 | (-0.28, 0.12) |  | -0.04 | (-0.21, 0.13) |  |
| Individual cohort models also adjusted for maternal age at delivery (continuous), maternal education (categorical), parity (categorical), maternal exposure to tobacco (HOME Study: cotinine level (continuous) and CCAAPS: Non-smokers and Smokers (categorical), and weight gain during pregnancy (continuous). HOME model also adjusted for maternal pre-pregnancy BMI (continuous). Pooled sample models adjusted for cohort (categorical), maternal age at delivery (continuous), maternal education (categorical), parity (categorical), maternal exposure to tobacco (categorical: non-smokers and smokers), and weight gain during pregnancy (continuous).  Abbreviations: ECAT, Elemental carbon attributable to traffic; HOME, Health Outcomes and Measures of the Environment; CCAAPS, Cincinnati Childhood and Air Pollution Study. | | | | | | | | | |

**
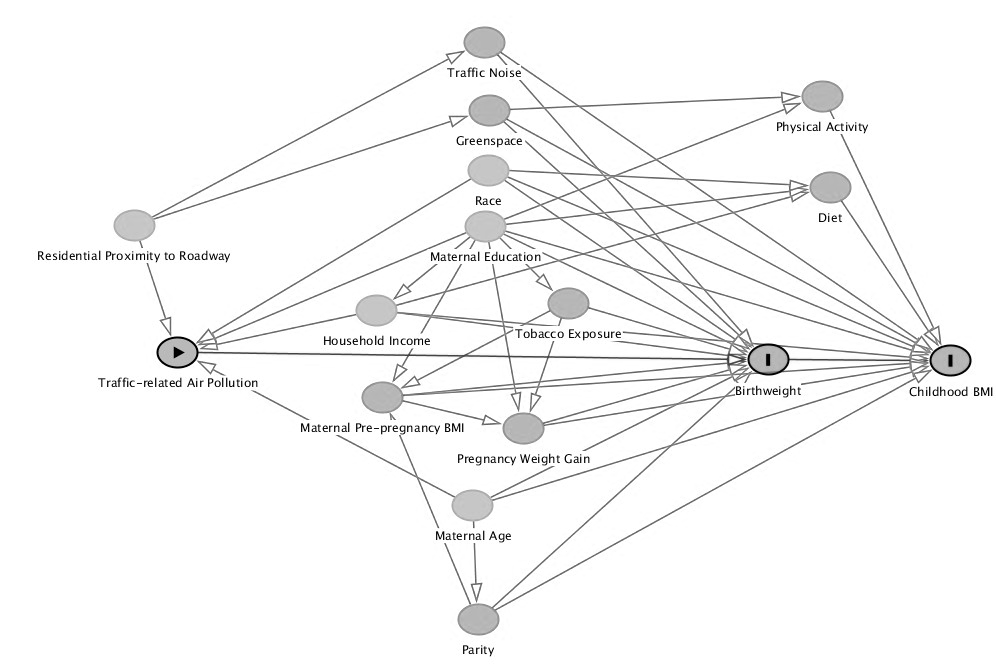
**

**Supplemental Digital Content 11. Directed acyclic graph of variables potentially confounding the association of ECAT with birthweight and childhood BMI**
